# Uterine Contractions’ Pattern in Active Phase of Labor as a Predictor of Failure to Progress

**DOI:** 10.5539/gjhs.v6n3p200

**Published:** 2014-03-24

**Authors:** Tahereh Galini Moghaddam, Nargess Moslemizadeh, Zahra Seifollahpour, Zohreh Shahhosseini, Mahmonir Danesh

**Affiliations:** 1 Mazandaran University of Medical Sciences, Sari, Iran

**Keywords:** failure to progress, uterine contraction, fall / rise ratio

## Abstract

**Background:**

Failure to progress remains a key indication for cesarean section which caused by different factors including uterine contractions. If it is diagnosed in the primary phase of labor, a better prognosis can thus be made. The purpose of this study was to find a possible correlation between pattern of uterine contraction and progression of labor.

**Method:**

During this study, 120 women referred for delivery to an educational hospital’s maternity ward in the North of Iran in 2010 were included. Uterine contractions of mothers were recorded in dilatation of 4 to7 cm for an hour. In this way, F/R ratio which means the time that a contraction needs to return from its peak to baseline (Fall) divided to the time for a contraction to rise to its peak (Rise) was calculated. All of the participants were followed until delivery, vaginal delivery or caesarean section.

**Results:**

Mean and standard deviation of fall to raise ratio was 1.54 ± 0.26 in mothers with vaginal delivery versus 1.74 ± 0.21 for others underwent caesarean section (OR = 0.44, 95% CI: 0.005- 0.42, P < 0.001). Sensitivity, specificity, and predictive values (positive and negative) of mentioned ratio were 68.32%, 70.01%, 69.55%, and 68.91%, respectively.

**Conclusion:**

By considering acceptable predictive value of uterine contractions’ pattern in the active phase of labor, it could help to timely diagnosis of failure to progress and consequently suitable intervention which probably maintain better health of both mother and fetus.

## 1. Introduction

Failure to progress is the most important indication for cesarean section and represents 25% of all emergency cesarean sections in primigravida women (Danforth & Gibbs, 2008; Kumari & Thomas, 2012). This problem is developed when a large size of fetal head or unsuitable position between the fetus’ head and mother’s pelvis diameter as well as mismatch between two these components exists, which is named cephalo-pelvic disproportion (CPD) in the field of obstetrics (Gao et al., 2013; Kovavisarach & Buddeewong, 2012). CPD inhibits progress of labor which in turn increases the chance of maternal and fetal complications (Hodnett, Gates, Hofmeyr, Sakala, & Weston, 2011; Jongen, Halfwerk, & Brouwer, 1998). Some of these complications include: the risk of chorioamnionitis, birth trauma, postpartum hemorrhage and infection as well, which in the cases of forceps or vacuum delivery, these complications could be increase. On the other hand, fetal complications due to CPD include fetal distress, bleeding and increased intracranial cerebral palsy and an increasing in fetal death rates (Chen, Uryasev, & Young, 2004; Shields, Ratcliffe, Fontaine, & Leeman, 2007; Tsvieli, Sergienko, & Sheiner, 2012).

Many studies have been conducted to predict CPD at first stages of labor (Benjamin, Daniel, Kamath, & Ramkumar, 2012; Harper, Odibo, Stamilio, & Macones, 2013; Liselele, Boulvain, Tshibangu, & Meuris, 2000; Macones et al.,2013). The aim of these diagnostic procedures is to evaluate the capacity of mothers’ pelvic in accordance to estimated fetal weight which was determined by ultrasound. Besides, some formulates are used to anticipate CPD based on the ratio of fetal size to mothers’ pelvic diameter. Due to poor predictive value of these methods, they are less applicable (Ferguson II et al., 1998; Spörri et al., 2002). It’s meanwhile, in a successful vaginal delivery, there are three factors including: position and size of the fetus (as passenger), maternal pelvic dimensions (as passage) and uterine contractions (as power) (Cunningham, 2009). Studies performed on diagnosis of CPD have so far focused on the two first factors whereas the third factor, uterine contractions, is given less attention.

Over all, two types of uterine contraction disorders such as hypotonic and hypertonic contractions exist. In the hypotonic disorder regardless hypertonic uterine dysfunction, in the active phase of labor (dilatation > 4 cm), the base tonicity of uterus is not increased. In addition in the cases of hypertonic contractions, due to lack of harmony in the impulses which root from one or both cornea and because the contractions of the middle segment of the uterus are more powerful from fundal contractions’ force, effective contractions during labor are absent (Cunningham, 2009; Shields et al., 2007; Savitsky et al.,2013).

Pattern of uterine contractions can be evaluated using external monitoring as well as internal. With the start of contractions and increase of intrauterine pressure, the height of the contraction curve increases and decreases following reduced intrauterine pressure (Gonçalves, Pinto, Ayres-de-Campos, & Bernardes, 2014). One landmark for evaluating quality of a contraction is F/R ratio which means the time that a contraction needs to return from its peak to baseline (Fall) divided to the time for a contraction to rise to its peak (Rise). In the case of a CPD, the interval between contractions increases and the height of the contraction curve is reduced, a warning sign for re-evaluating the patient.

Efforts in determining the factors that could anticipate CPD before failure to progress in labor and consequential complications has led many researchers to study the pattern of uterine contractions during delivery. The aim of this study was to determine the predictive value of uterine contractions in the active phase of labor to provide suitable criteria for a precise prediction of failure to progress.

## 2. Methods

This diagnostic study was done in 2010 on 120 women referred for delivery to an educational hospital’s maternity ward in Mazandaran province, in the North of Iran. Inclusion criteria were: null parity, singleton pregnancy, normal cephalic presentation, lack of inherited defaults in the fetus, non-use of magnesium sulfate, absence of macrosomia and normal diameters of mother’s pelvis. Pelvic examination for all of the mothers were done and if diagonal conjugate was equal or greater than 11.5 cm, the lateral pelvic walls was converging and the pubic angle arc was equal or more than 90°, the pelvic diameters were considered favorable. Excluding criteria were mothers who underwent cesarean delivery due to other indications, except failure to progress and CPD.

In the next step, uterine contractions of volunteers’ pregnant women in the maximum slope of active phase of labor and dilatation of 4 to 7 cm were monitored continuously an hour by a Chinese external monitoring machine, named BISTUS. During this period the pattern of uterine contractions were recorded and F/R ratio was calculated in each contraction. For all of the participants, progress of labor was monitored using Friedman graph. When drawing curves fell below the alert line, the mothers’ contractions were assessed and if contractions were inadequate, oxytocin infusion was established and if the registered curve was under the action line, a cesarean section was performed. Otherwise, mothers monitored until vaginal delivery except when other obstetrical indications of caesarean delivery were presented. Data gathering were continued until 120 mothers were included in either vaginal delivery group (60 cases) or caesarean delivery group (60 cases). This sample size was considered in attention to confidence level of 95 % and power of 90% for this study.

For eligible participants a check list of demographic and obstetrical characteristics was completed included age, pre-pregnancy body mass index, gestational age, results of pelvic diameters, use of oxytocin, cervical dilatation and effacement, fetal station and fetal membranes.

The collected data were coded and analyzed using the Statistical Package for Social Sciences for Windows version 16.0 (SPSS Inc., Chicago, IL, USA). Means and standard deviations were computed and reported. Also t-test, Chi square, Pearson correlation coefficients and Logistic Regression were used to analytical analysis. Finally to determine the diagnostic value of F/R ratio, the area under the receiver operating curve was used. In this regard, P value < 0.05 was considered statistically significant.

This study was approved by ethical committee of Research Vice Chancellors of Mazandaran University of Medical Sciences. All of the participants were informed of the purpose and design of the study as well as confidentiality of gathered data. They have right to withdraw the study at any time and they provided written informed consent before the beginning of study. Also, permission for data collection was obtained from the Area University Chief Executive Officers.

## 3. Results

The findings showed the mean of participants’ age in vaginal delivery group were 23.02 ± 3.22 vs. 25.23±4.48 in the cesarean delivery group (p= 0.002). Some of the demographic and obstetrical characteristics of participants have shown in Table 1. Also it’s found that the results of pelvic examination in both vaginal and cesarean delivery groups were different not significantly (Table 2).

**Table 1:** Demographic and obstetric characteristics of participants in vaginal and cesarean delivery groups

		Vaginal delivery group (n=60)	cesarean delivery group (n=60)	P value
Maternal age (Years)*		23.02 ± 3.22	25.23 ± 4.48	0.002
Gestational age (Weeks)*		38.97 ± 0.98	39.58 ± 1.11	0.002
BMI before pregnancy (Kg/m^2^)*		26.88 ± 2.71	27.54 ± 1.99	0.132
Fetal weight(gr)*		3259.49 ± 322.97	3447.83 ± 789.21	0.002
Cervical dilatation(Cm)*		5.15 ± 1.05	4.93 ± 0.95	0.240
Cervical effacement(Percent)*		61.17 ± 9.58	58.17 ± 8.73	0.761
Station**	-3	21(35.00)	35(58.32)
	-2	24(40.00)	20(33.33)	
	-1	13(21.67)	5(8.35)	0.024
	0	2(3.33)	0(0)	
Fetal membrane**	Intact	29(48.33)	39(65.00)	
	Ruptured	31(51.67)	21(35.00)	0.048
Apgar score**	<7	1(1.66)	0(0)
	>7	59(98.34)	100(100)	0.315
Infusion of Oxytocin**	Yes	30(50)	36(60)
	No	30(50)	24(40)	0.170

* Mean±SD

** Number (Percent)

**Table 2:** Characteristics of participants in vaginal and cesarean delivery groups based on pelvic examination

		Vaginal delivery group (n=60)	cesarean delivery group (n=60)	P value
Inlet pelvis*	Favorable	60(100)	60(100)	1.00
	Unfavorable	0(0)	0(0)	
Mid pelvis*	Favorable	58(96.66)	55(91.66)	0.219
	Unfavorable	2(3.34)	5(8.34)	
Outlet pelvis*	Favorable	60(100)	58(96.66)	0.248
	Unfavorable	0(0)	2(3.34)	

* Number (Percent).

To the base of this study, the average of F/R ratio in the vaginal delivery group was 1.54 ± 0.26 compared to cesarean delivery group 1.74 ± 0.21 (P < 0.001). Logistic regression has showed a statistically significant relationship between F/R ratio and failure to progress (OR = 0.44, 95% CI: 0.005- 0.42, P < 0. 001). Also Pearson correlation coefficient has found relationship between F/R ratio and maternal age (r = 0.188, P < 0.001), gestational age (r = 0.193, P < 0.001), fetal weight (r = 0.352, P < 0.001).

Another finding toward predictive value of F/R ratio in failure to progress showed the sensitivity and specifity of test were 68.32% and 70.01% respectively. In this regard, the positive and negative predictive value was 69.55% and 68.91% accordingly. The area under the receiver operating characteristics curve was 0.747 with a cut-off point of 1.68.

## 4. Discussion

This study has shown that there is a relationship between the F/R ratio and cesarean delivery due to failure to progress. Higher average of F / R ratio in the cesarean delivery group compared to vaginal delivery group (1.74 vs 1.54) is in accordance with some studies which have shown if the F / R ratio is greater than 1.68, the number of cesarean sections due to failure to progress increases (Althaus et al.,2006).

Findings of this study in line with other studies have proved that increasing gestational age is associated with failure to progress of labor (Althaus et al., 2006; Khunpradit, Patumanond, & Tawichasri, 2005). Although in a study conducted by Oppenheimer, no relationship has been found between two these factors (Oppenheimer et al.,2002). It seems by increasing gestational age and subsequently fetal weight gain, the increased obstetric interventions lead to a higher probability of cesarean sections due to failure to progress (Chen et al., 2004; Cunningham, 2009). The finding of this study toward a significant relationship between maternal age and failure to progress like other studies (Althaus et al., 2006; Bayrampour & Heaman, 2010) may root in this issue that some uterine anomalies like uterine fibroma and cervical fibrosis increase with maternal age which in turn causes irregularity of uterine contractions and resulted in failure to progress (Cunningham, 2009). Despite these findings in some studies no association was found between maternal age and failure to progress (Chen et al., 2004; Khunpradit et al.,2005). In this study, in accordance of some studies there was a relationship between fetal weight and failure to progress (Althaus et al., 2006; Oppenheimer et al.,2002). It’s speculated fetal head circumference increases with fetal weight gain and then cephalo-pelvic disproportion increases, particularly in cases where the mother’s pelvis is relatively narrow (Chen et al.,2004). This study has proven that, when recording the contractions, the higher the fetal station, the higher the probability of lack of failure to progress. It seems if the fetal head is not engaged during the active phase of labor, failure to progress in labor will associate with primigravid women (Gilboa et al.,2013).

In conclusion this study showed that CPD influences uterine contractions and will causes longer contraction settling times. What factors are causing these changes in uterine contractions is still unknown that can be investigated in future studies. It’s noticeable an issue that is important in the progression of delivery process, in addition to the duration, number and pattern of the uterine contraction, is the intensity of the contraction, which is a major factor that should be noted when evaluating uterine contractions. Thus by considering acceptable predictive value of uterine contractions’ pattern in the active phase of labor, it could help to timely diagnosis of failure to progress and consequently suitable intervention which probably maintain better health of both mother and fetus. Finally, the results of this study have implications for policy and practice. This study emphasizes the importance of a health care providers’ preparation to manage a pregnant mother from the moment she arrives in labor room until delivery. In this way, if the providers are able to understand the risky signs of an abnormal labor and established timely interventions, the safe motherhood is anticipated.

From limitation of this study were limited facilities for internal monitoring in Iran. Such that only the curve of the uterine contraction was used in this study and the intrauterine pressure was not measured in order to further evaluation of the quality of the uterine contractions. As studies performed on the pattern of uterine contractions are very limited, if there are more possibilities and better equipment in future studies, it’s anticipated more relationships could be found.

## References

[ref1] Althaus J. E, Petersen S, Driggers R, Cootauco A, Bienstock J. L, Blakemore K. J (2006). Cephalopelvic disproportion is associated with an altered uterine contraction shape in the active phase of labor. American journal of obstetrics and gynecology.

[ref2] Bayrampour H, Heaman M (2010). Advanced maternal age and the risk of cesarean birth: a systematic review. Birth.

[ref3] Benjamin S. J, Daniel A. B, Kamath A, Ramkumar V (2012). Anthropometric measurements as predictors of cephalopelvic disproportion. Acta Obstetricia et Gynecologica Scandinavica.

[ref4] Chen G, Uryasev S, Young T. K (2004). On prediction of the cesarean delivery risk in a large private practice. American journal of obstetrics and gynecology.

[ref5] Cunningham F. G (2009). Williams obstetrics.

[ref6] Danforth D. N, Gibbs R. S (2008). Danforth’s obstetrics and gynecology.

[ref7] Ferguson J, Newberry Y. G, DeAngelis G. A, Finnerty J. J, Agarwal S, Turkheimer E (1998). The fetal-pelvic index has minimal utility in predicting fetal-pelvic disproportion. American journal of obstetrics and gynecology.

[ref8] Gao Y, Xue Q, Chen G, Stone P, Zhao M, Chen Q (2013). An analysis of the indications for cesarean section in a teaching hospital in China. European Journal of Obstetrics & Gynecology and Reproductive Biology.

[ref9] Gilboa Y, Kivilevitch Z, Spira M, Kedem A, Katorza E, Moran O, Achiron R (2013). Head progression distance in prolonged second stage of labor: relationship with mode of delivery and fetal head station. Ultrasound in Obstetrics & Gynecology.

[ref10] Gonçalves H, Pinto P, Ayres-de-Campos D, Bernardes J (2014). External Uterine Contractions Signal Analysis in Relation to Labor Progression and Dystocia. XIII Mediterranean Conference on Medical and Biological Engineering and Computing 2013. IFMBE Proceedings.

[ref11] Harper L. M, Odibo A. O, Stamilio D. M, Macones G. A (2013). Radiographic Measures of the Midpelvis to Predict Cesarean. American journal of obstetrics and gynecology.

[ref12] Hodnett E. D, Gates S, Hofmeyr G. J, Sakala C, Weston J (2011). Continuous support for women during childbirth. Cochrane Database of Systematic Reviews.

[ref13] Jongen V, Halfwerk M, Brouwer W (1998). Vaginal delivery after previous caesarean section for failure of second stage of labour. BJOG: An International Journal of Obstetrics & Gynaecology.

[ref14] Khunpradit S, Patumanond J, Tawichasri C (2005). Risk indicators for cesarean section due to cephalopelvic disproportion in Lamphun Hospital. Journal-Medical Association of Thiland.

[ref15] Kovavisarach E, Buddeewong P (2012). Diagnosis of Cephalopelvic Disproportion or Failure to Progress of Labor in Rajavithi Hospital Compare with The Criteria of Royal Thai College of Obstetricians and Gynaecologists. Thai Journal of Obstetrics and Gynaecology.

[ref16] Kumari P. S, Thomas V (2012). A cross sectional study of rate and determinants of caesarean sections among mothers attending government maternity hospital, Hyderabad. Int J Med Pharm Sci.

[ref17] Liselele H. B, Boulvain M, Tshibangu K. C, Meuris S (2000). Maternal height and external pelvimetry to predict cephalopelvic disproportion in nulliparous African women: a cohort study. BJOG: An International Journal of Obstetrics & Gynaecology.

[ref18] Macones G. A, Jen Chang J, Stamilio D. M, Odibo A. O, Wang J, Cahill A. G (2013). Prediction of Cesarean Delivery Using Fetal-Pelvic Index. American journal of obstetrics and gynecology.

[ref19] Oppenheimer L. W, Bland E. S, Dabrowski A, Holmes P, McDonald O, SHI W. W (2002). Uterine contraction pattern as a predictor of the mode of delivery. Journal of perinatology.

[ref20] Savitsky A. G, Savitsky G. A, Ivanov D. O, Mikhailov A. V, Kurganskiy A. V, Mill K. V (2013). The myogenic mechanism of synchronization and coordination for uterine myocytes contractions during labor. Journal of Maternal-Fetal and Neonatal Medicine.

[ref21] Shields S. G, Ratcliffe S. D, Fontaine P, Leeman L (2007). Dystocia in nulliparous women. Am Fam Physician.

[ref22] Spörri S, Thoeny H. C, Raio L, Lachat R, Vock P, Schneider H (2002). MR imaging pelvimetry: a useful adjunct in the treatment of women at risk for dystocia?. American Journal of Roentgenology.

[ref23] Tsvieli O, Sergienko R, Sheiner E (2012). Risk factors and perinatal outcome of pregnancies complicated with cephalopelvic disproportion: a population-based study. Archives of gynecology and obstetrics.

